# Altered Gut Microbiota and Immunity Defines *Plasmodium vivax* Survival in *Anopheles stephensi*

**DOI:** 10.3389/fimmu.2020.00609

**Published:** 2020-05-14

**Authors:** Punita Sharma, Jyoti Rani, Charu Chauhan, Seena Kumari, Sanjay Tevatiya, Tanwee Das De, Deepali Savargaonkar, Kailash C. Pandey, Rajnikant Dixit

**Affiliations:** ^1^Laboratory of Host-Parasite Interaction Studies, ICMR-National Institute of Malaria Research, New Delhi, India; ^2^Bio and Nanotechnology Department, Guru Jambheshwar University of Science and Technology, Haryana, India

**Keywords:** *Anopheles stephensi*, midgut, microbiome, *Plasmodium vivax*, tripartite interactions

## Abstract

Blood-feeding enriched gut-microbiota boosts mosquitoes' anti-*Plasmodium* immunity. Here, we ask how *Plasmodium vivax* alters gut-microbiota, anti-*Plasmodial* immunity, and impacts tripartite *Plasmodium*-mosquito-microbiota interactions in the gut lumen. We used a metagenomics and RNAseq strategy to address these questions. In naïve mosquitoes, *Elizabethkingia meningitis* and *Pseudomonas* spp. are the dominant bacteria and blood-feeding leads to a heightened detection of *Elizabethkingia, Pseudomonas* and *Serratia 16S rRNA*. A parallel RNAseq analysis of blood-fed midguts also shows the presence of *Elizabethkingia-related* transcripts. After, *P. vivax* infected blood-meal, however, we do not detect bacterial 16S rRNA until circa 36 h. Intriguingly, the transcriptional expression of a selected array of antimicrobial arsenal cecropins 1–2, defensin-1, and gambicin remained low during the first 36 h—a time frame when ookinetes/early oocysts invaded the gut. We conclude during the preinvasive phase, *P. vivax* outcompetes midgut-microbiota. This microbial suppression likely negates the impact of mosquito immunity which in turn may enhance the survival of *P. vivax*. Detection of sequences matching to mosquito-associated *Wolbachia* opens a new inquiry for its exploration as an agent for “paratransgenesis-based” mosquito control.

## Introduction

A blood meal is an essential requirement for the reproductive success of adult female mosquitoes. Immediately after blood meal uptake, mosquitoes' gut physiology undergoes complex modulation to facilitate rapid blood meal digestion and activation of the vitellogenesis process (1, 2). The blood-meal also triggers proliferation of gut microbiome eliciting immune response (3, 4), and once the blood meal digestion is completed within first 30 h, the immune response apparently ceases to basal level (5, 6).

This mosquito's gut immune response may indirectly affect the early development of *Plasmodium* when mosquitoes take infected blood (7–9). Removal of gut microbes by antibiotic treatment enhances *Plasmodium* survival, however, our understanding of how *Plasmodium* manages its safe journey to the gut and succeeds to develop in the susceptible mosquitoes remains unclear (10). A tripartite interaction of gut-microbes-parasites during earlier or pre-invasive phase of the malaria infection is expected to play a vital role in the success of the parasite's journey through the gut lumen (11–15). But a great deal of understanding that how a parasite manages its survival during acute gut-microbe interaction is still limited (4). Once the gut epithelial is invaded, the *Plasmodium* population undergoes several bottlenecks reducing the oocysts load either to zero in naturally selected refractory mosquito strains, or a few oocysts in a susceptible mosquito vector species (16, 17).

Within 8–9 days post-infection, the surviving oocysts rupture to millions of sporozoites, released in the hemolymph (11). During free circulation, sporozoites compete to invade the salivary glands, and if not successful are rapidly cleared by the mosquito immune blood cells “hemocytes” (6, 16–18). The invaded sporozoites reside in clusters in the salivary glands till they get a chance to invade the vertebrate host (19, 20). Though studies targeting individual tissues such as midgut or salivary glands are valuable, the genetic basis of *Plasmodium* population alteration is not well-understood (21).

**Figure 1:**
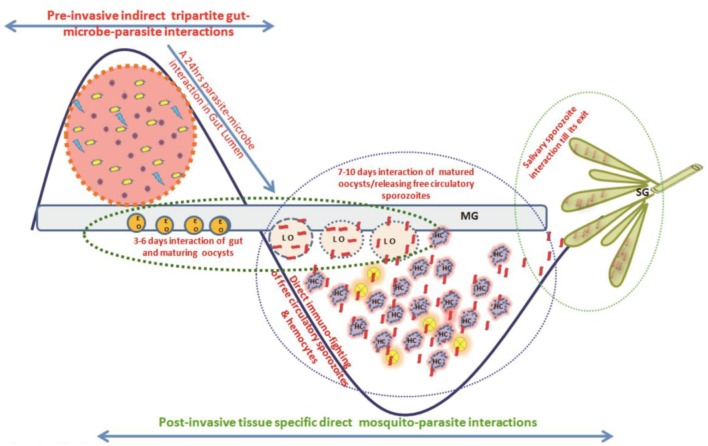
A proposed working hypothesis to decode a system-wide pre and post-gut invasive phases of *P. vivax-*mosquito interactions: Immediately after an infected blood meal, sexual developmental physiology of *Plasmodium* rapidly change to adapt mosquitoes' hostile gut-lumen environment and progressively faces gut-microbiota boosted anti-*Plasmodium* immunity. Though the mechanism that how *Plasmodium* manages safe journey and survival from gut lumen 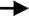 gut epithelium 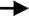 hemolymph 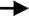 salivary gland 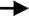 vertebrate host is not fully known, but we propose and decode (i) a 24–30 h of pre-invasive phase of an indirect gut-microbe-parasite interaction in the gut lumen for ookinetes invasion; and (ii) a longer post-gut invasive, a direct parasite-tissues such as midgut (MG), hemocyte (HC), and salivary gland (SG) interactions, are crucial for the *Plasmodium* survival (22). Schematically, 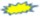, represents *Plasmodium* gametocytes; 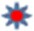 and 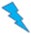, different bacterial species; 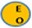, the mustard yellow circle represents early gut invaded maturing oocysts (EO); 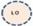, peach circle with blue dotted boundary is Late rupturing oocysts (LO); 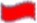, red ribbon is sporozoite; 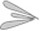, salivary lobes; 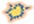, the purple cloudy structure is hemocyte.

We hypothesized that for its survival *Plasmodium* must overcome at least two levels of competitive challenges (Figure 1). The first one follows a 24–30 h pre-invasive phase of interaction initiated immediately after a blood meal influencing: (a) parasite development and adaptation to physiologically distinct but hostile gut environment than vertebrate host; (b) nutritional resources competition against exponentially proliferating gut microbes, and (c) the barrier(s) infringement of gut epithelial prior maturation of peritrophic matrix, a unique but unresolved mechanism of self-protection. A second phase follows post-gut invasion of ookinetes which encompasses a direct interaction of (d) developing and maturing oocysts within midgut (8–10 days); (e) free circulatory sporozoites and hemocytes; and (f) salivary invaded sporozoites within salivary glands (10–16 days).

Thus, to decode the tissue-specific molecular complexity/nature of interactions, we designed and carried out a system-wide investigation. In this report, we followed changes (1) in the gut microbiota under naïve, blood-fed and *Plasmodium* infected blood fed conditions, and (2) changes in the expression of selected immune markers. Our data demonstrates how an early suppression of gut microbiome proliferation, and hence gut immunity may support *P. vivax* survival during the pre-invasive phase of development. While in the second complimentary report, we demonstrate that post-gut invasion, a smart molecular relationship with individual tissues such as midgut, hemocytes, salivary glands, and strategic changes in the genetic makeup of *P. vivax* favor its survival in the mosquito host [see (22)].

## Materials and Methods

Technical overview presented in Figure S1.

### Mosquito Rearing

*Anopheles stephensi* colonies were reared in the central insectary facility at ICMR-National Institute of Malaria Research (NIMR). A constant 28 ± 2°C temperature and relative humidity of ~80% was maintained in the insectarium. A live rabbit was offered as a blood meal for egg maturation and gonotrophic cycle maintenance (6, 23).

### Metagenomic Study

#### Tissue Dissection and Sample Preparation

For the study, *A. stephensi* pupae (*n* = 200) were reared in ethanol sterilized plastic cages fitted with autoclaved mesh cloth on the top. Ten percent sterile, fresh sugar solution was provided daily with a sterile cotton swab fitted in a test tube throughout the experiment. For metagenomics studies, we collected the guts from 4 to 5 days old either sugar-fed or blood-fed ~50 adult female mosquitoes. Dissections were performed after surface sterilization of the mosquitoes using 75% ethanol for 1 min in 50 μl 1X Saline-Tris-EDTA (STE) buffer. Total DNA from pooled gut samples was extracted under aseptic conditions of the laminar airflow, as described earlier (24). In brief, the tissue was homogenized using handheld battery run homogenizer and contaminating protein was digested by proteinase K treatment. For DNA quality assessment ~5 μl of gDNA was loaded on 0.8% agarose gel and run through standard agarose gel electrophoresis to visualize the single intact band as the quality mark (Figure S2). Quantification was performed using Qubit dsDNA BR Kit (Thermo Fisher Scientific Inc.) after checking the A_260/280_ ratio of 1 μl of each sample using Nanodrop 8000.

#### 16S rRNA Based Metagenomic Sequencing and Analysis

Using Nextera XT Index Kit (Illumina Inc.), the amplicon libraries were prepared from the qualified DNA samples. Primers were designed and synthesized using the V3-V4 hyper-variable region of 16S rDNA gene (Table ST1). The Illumina adaptors ligated amplicons were amplified by using i5 and i7 primers for multiplex indexing. Purification of the amplicon libraries was performed on 1X AMpureXP beads and checked for its quality with Bioanalyzer 2100 Agilent using a DNA1000 chip and quantification was done on fluorometer by Qubit dsDNA HS Assay kit (Life Technologies) (Figure S3). A Paired-End (PE) sequencing was done with MiSeq technology and generated data was stitched into single-end reads. Final clean reads were subjected for Operational Taxonomic Units (OTUs) clustering and analysis using Quantitative Insights into Microbial Ecology (QIIME version 1.9.1) software package comprising of tools and algorithms such as FastTree for heuristic based maximum-likelihood phylogeny inference (25). The taxomic assignment to the final OTUs was done by RDP classifier data using a naïve Bayesian classifier, raw data output as .biom files were further analyzed through MEGAN software (26).

#### Gut RNAseq Analysis

Approximately one microgram purified total RNA from pooled 24–48 h post-blood-fed ~20 adult female mosquitoes guts, was subjected to double-stranded cDNA library preparation (Clontech SMART^TM^) and sequencing (Illumina Technology), as described earlier (23, 27–29). Briefly, the purified ds cDNA sample (~200 ng) was sheared using the Covaris sonication method and the overhangs so generated were end-repaired before further processing. The paired-end cDNA libraries were generated through Illumina TruSeq Nano DNA HT Library Preparation Kit using 2 × 150 PE chemistry on NextSeq for generating ~1 GB data as per the described protocol. The end-repaired fragments were subjected to enrichment by a limited number of PCR cycles after adding a poly A-tail and adapter ligation. Library quantitation and qualification were performed using DNA high Sensitivity Assay Kit. The sequencing of whole transcriptomes was performed on Illumina NextSeq. Trimmomatic v0.30 software was used to filter the raw reads. After removing adaptor sequences and low quality (QV <20) reads, high-quality clean reads were used to make *de novo* assembly using Trinity software (release r2013-02-25). CD-HIT-EST (Version 4.6) was used to remove the shorter redundant transcripts. All CDS were predicted from transcript using Transdecoder and selected longest frame transcripts were subjected for functional annotation using BLASTX against NR database and BLAST2GO program [see also (22)].

#### Artificial Membrane Feeding and *P. vivax* Infection

The collection of the *P. vivax* infected patients' blood samples was approved by the Ethics committee of NIMR, Delhi (ECR/NIMR/EC/2012/41). Prior collection of blood samples, a written informed consent (IC) was obtained from donors visiting to institutional clinic. Venous blood was drawn into heparin-containing tubes and kept at 37°C till feeding. Overnight starved 4–5 days old female *A. stephensi* mosquitoes were fed using pre-optimized artificial membrane feeding assay (AMFA). Only full-fed mosquitoes were maintained at optimal insectarium conditions and positive infection was confirmed by standard mercurochrome staining of gut oocysts readily observed under a compound microscope. Desired tissue samples such as midgut, salivary glands, hemocytes were collected from ~20 infected or uninfected adult female mosquitoes for subsequent analysis as reported in Tevatiya et al. (22). However, we excluded mosquito samples which showed poor/negative oocysts development in their gut.

#### RNA Isolation and Differential Gene Expression Analysis

Total RNA from different tissues was isolated (30) from naïve, blood-fed or *Plasmodium*-infected *A*. *stephensi* mosquitoes (*n* = 20) and cDNA was synthesized using Verso cDNA synthesis kit (Thermo Fisher Scientific, #AB1453A) as per manufacturer protocol. Routine laboratory optimized RT-PCR and agarose gel electrophoresis processes were followed for differential expression of the selected genes. Relative gene expression was performed by QuantiMix SYBR green dye (Thermo scientific 2X DyNAmo Color Flash Sybr Green Master Mix Cal. No. F-416) in Eco-Real Time (Illumina, USA; Cat. No. EC-101-1001) or CFX-96 (Biorad, USA), Real-Time PCR machine. PCR cycle parameters included initial denaturation at 95°C for 15 min, followed by 44 cycles of 10 s at 95°C, 20 s at 55°C, and 22 s at 72°C with a final extension of 15 s at 95°C, 15 s at 55°C, and 15 s at 95°C. All qPCR measurements were performed with two technical replicates to rule out any possibility of biases. At least three independent biological replicates were tested for better evaluation. Differential gene expression was evaluated using the *ddCT* method and statistically analyzed by the student “*t”* test. List of primers presented in the Table ST2.

## Results

### *Elizabethkingia* and *Pseudomonas* Predominate Mosquito Gut

**Figure 2:**
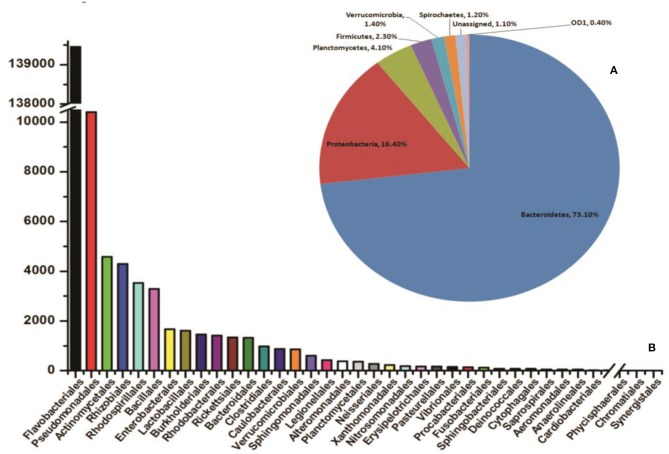
QIIME analysis based gut microbial community structure in naïve mosquitoes (*n* = 40). **(A)** Diversity of the microbiota in the mosquito gut, at phylum level showing dominant association of Bacteroidetes and Proteobacteria followed by Planctomycetes; Firmicutes, Verrucomicrobia, Spirochaetes; **(B)** The bar graph representing abundance of different bacteria at order level based on number of gut metagenomics reads, where Flavobacteriales and Pseudomonadales are the most abundant orders to which *Elizabethkingia* and *Pseudomonas* belong, respectively. Enterobacterales are also among the top ten abundant orders which include Serratia.

To identify and catalog gut-associated bacteria, we sequenced and analyzed a total of 3,68,138 Illumina raw reads originating from naive mosquito gut metagenomic library. Diversity richness indices such as Shannon-Weaver (1.662 ± 0.02) and Simpson reciprocal (1.667 ± 0.001) showed an optimal estimation and even distribution of species. A QIIME analysis at phylum level showed that mosquitoes gut dominantly harbors Bacteroidetes (73.13%); Proteobacteria (16.4%); Planctomycetes (4.10%); Firmicutes (2.3%), Verrucomicrobia (1.4%), Spirochaetes (1.2%), OD1 (0.4%), and 1.10% 16S reads remained unassigned (Figure 2A). At the class level, the Flavobacteria and Gammaproteobacteria where *Elizabethkingia meningoseptica* and *Pseudomonas* sp. were the most abundant gram-negative bacteria, respectively (Figure 2B).

### Blood Meal Alters the Gut Microbiome Community Structure

**Figure 3:**
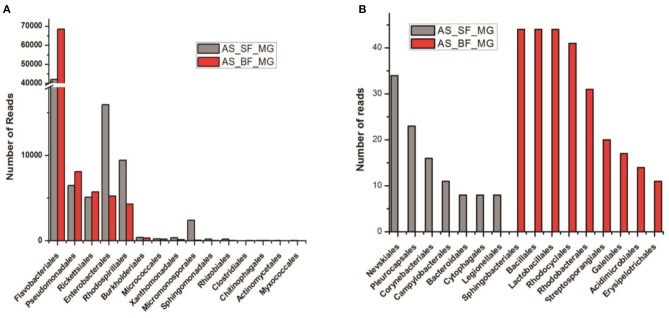
Blood-meal alters gut community structure (order level). **(A)** Comparative analysis of common bacterial community abundances among naïve and blood-fed mosquitoes gut (*n* = 50); **(B)** bar graph represents unique bacterial orders showing association with either sugar-fed (SF) or blood-fed (BF) mosquitoes gut. AS, *A. stephensi*.

In coherence with previous studies, we also observed that blood meal gradually enriched the total bacterial population in the gut till 24 h, which restored to their basal level within 48 h of blood meal (Figure S4). To further clarify that how blood meal influences individual bacterial population we cataloged and compared gut microbiome of naïve and 24 h blood-fed adult female mosquito guts. Alpha-diversity rarefaction curves estimate the full extent of phylotype richness and quantifiable diversity estimation (Figure S5). A normalized read count data comparison showed that blood meal not only enriched gut-associated dominant Flavobacteria but also favored modest enrichment of unique bacteria such as Bacillales, Lacto-bacillales, Spinghobacteriales, Rohocyclales (Figures 3A,B).

**Figure 4:**
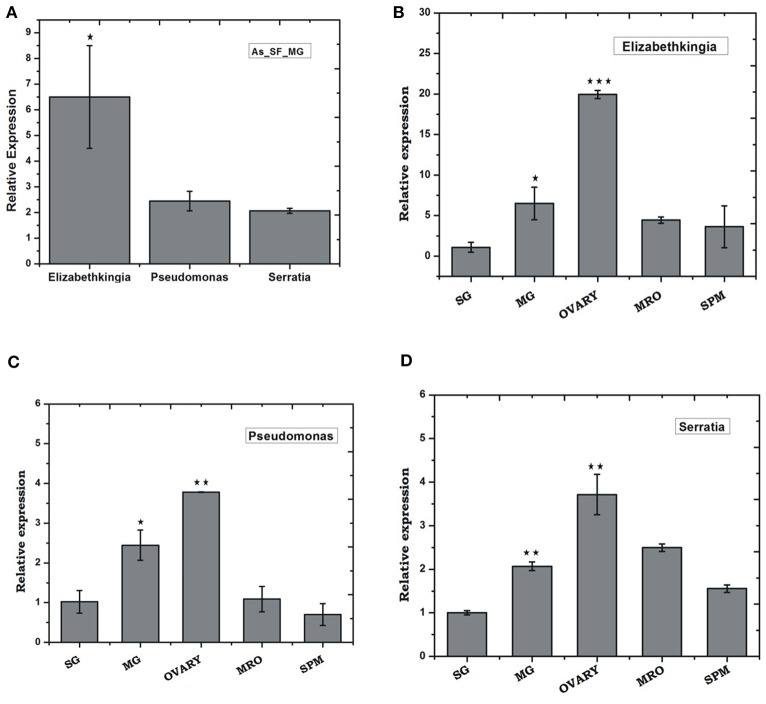
Tissue-specific relative distribution of dominant endo-symbiotic bacteria in the naïve mosquitoes: **(A)** Relative abundance of *Elizabethkingia* (*p* ≤ 0.05), *Pseudomonas, Serratia* in the naïve mosquito gut; tissue-specific relative abundance of **(B)** *Elizabethkingia* (MG *p* ≤ 0.033; Ovary *p* ≤ 0.00045); **(C)** *Pseudomonas* (MG *p* ≤ 0.025; Ovary *p* ≤ 0.0026); and **(D)** *Serratia* (MG *p* ≤ 0.0025; Ovary *p* ≤ 0.0072); AS, *A. stephensi*; SF, sugar fed; SG, salivary gland; MG, midgut; MRO, male reproductive organ; SPM, Spermathecae. Data was statistically analyzed considering SG expression as control sample for “*t*” test. *p < 0.05; **p < 0.005; ***p < 0.0005.

**Figure 5:**
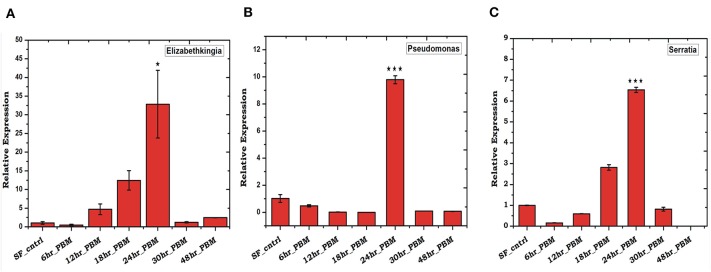
Blood feeding and species-specific distribution of gut microbes: Time-dependent relative abundance of **(A)** *Elizabethkingia* (*p* ≤ 0.001); **(B)** *Pseudomonas* (*p* ≤ 0.0005); and **(C)** *Serratia* (*p* ≤ 0.0001); in the blood-fed mosquitoes gut. The gut tissue was collected at different time intervals of 6, 12, 18, 24, 30, and 48 h post-blood-feeding. Data were statistically analyzed using student “*t”* test, where naïve sugar fed (SF) mosquito gut samples were considered as control against selected test sample. *p < 0.05; ***p < 0.0005.

To validate the above observation, we examined relative abundances of selected bacterial species, by Real-time PCR assay, using bacterial species-specific primers (see Table ST2). We observed a relatively higher abundance of bacteria such as *Pseudomonas, Elizabethkingia*, and *Serratia*, in the ovary and midgut than other tissues. However, within midgut, *Elizabethkingia* showed higher abundance than the *Pseudomonas* and *Serratia*, corroborating the metagenomic data (Figures 4A–D, Table ST3). Individual bacterial species such as *Elizabethkingia* (Flavobacteria), *Pseudomonas* and *Serratia* (Enterobacteriaceae) also showed a gradual enrichment until 24 h post-blood-feeding. However, post 30 h blood meal digestion the bacterial population restored to the basal level of naïve mosquito midgut (Figures 5A–C).

### RNAseq Recovers Molecular Signatures of Gut-Microbe Interaction

**Figure 6:**
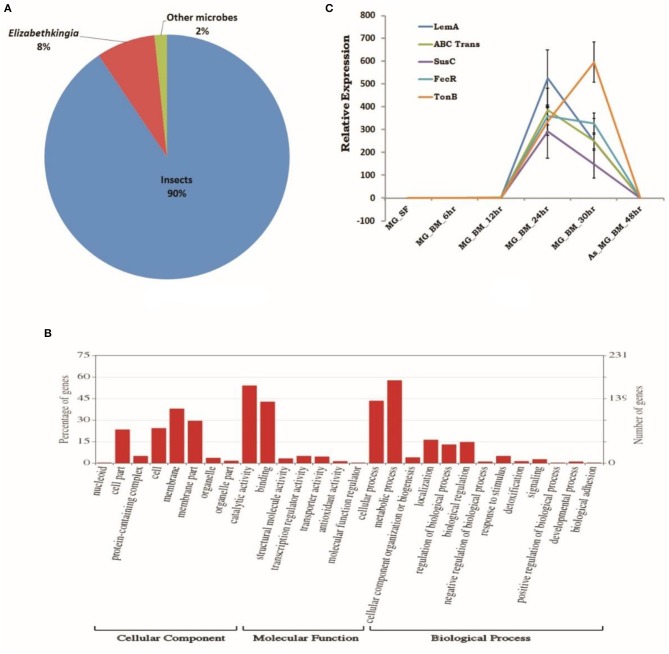
RNAseq identified microbial transcripts signatures. **(A)** Pie chart showing species distribution analysis of gut RNAseq data identifying transcripts having BLASTX homology to Insects (90%), *Elizabethkingia* as dominant gut endosymbiont bacteria (8%), and other microbes (2%); **(B)** molecular catalog of identified EK transcripts; and **(C)** transcriptional profiling of bacterial EK specific transcripts in response to blood meal.

To establish a molecular/ functional relation of gut-microbe interaction, we analyzed a total of 46,73,408 Illumina reads originating from 24 h post blood-fed gut RNAseq library (Table ST4). Surprisingly, a species distribution analysis of 5,041 full-length transcripts predicted that at least 90% of transcripts sequences matched to insects, but ~10% transcripts i.e., 479 CDS showed significant homology to microbial proteins (Table ST5). Transcripts homolog to insects dominantly matched to *A. gambiae* (~72%), *A*. *sinensis* (~13%), *A*. *darlingi* (~7%), *Aedes aegypti* (~1.6%), and *Culex* (~1.1%) (Figure 6A). A close examination of BLASTx analysis of microbial sequences/transcripts further identified that at least 8% of transcripts encode proteins homologous to *Elizabethkingia* (EK) (Figure 6A), strengthening our finding that EK constitutes a major gut endosymbiotic bacteria in *A. stephensi*. While remaining 2% of transcripts showed significant homology to other microbes such as *Annacalia alegera*; *Wolbachia* and viruses (Table ST5).

A comprehensive GO annotation of 391 putative transcripts indicated that EK bacterial species encodes the diverse nature of proteins (Figure 6B, Table ST5). Transcriptional profiling of selected bacterial transcripts encoding LEM A, Ton-B dependent receptor, FecR, ABC transporter, SusC/Rag family protein showed enriched expression in response to blood feeding and digestion (Figure 6C, Table ST6).

### Early *Plasmodium vivax* Infection Suppresses Gut Microbiota and Immunity

**Figure 7:**
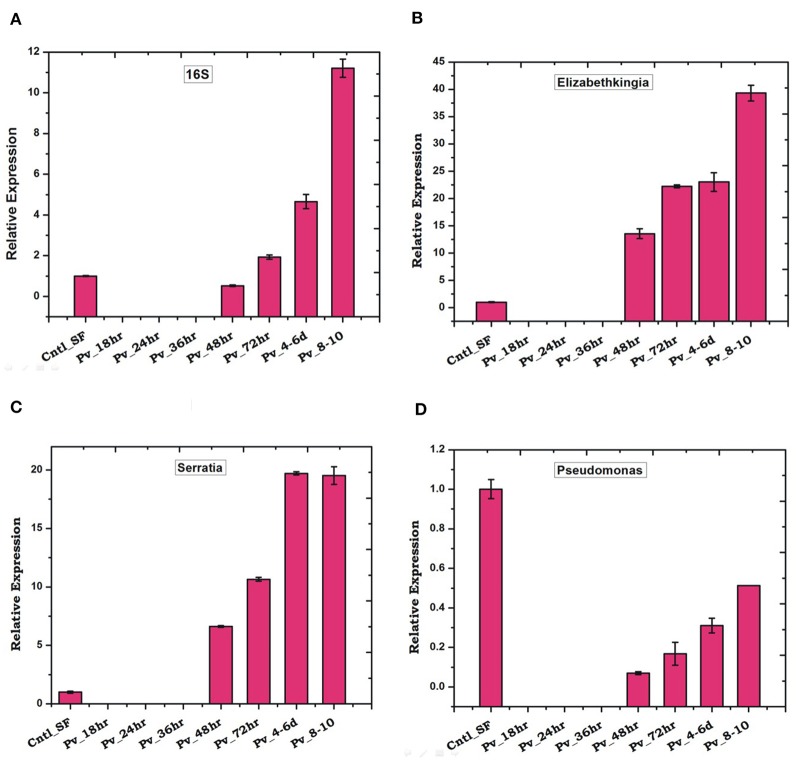
*P. vivax* infection cause early suppression and late restoration/enrichment of gut bacterial population: A time dependent relative quantification of gut microbiota in response to *Plasmodium vivax* (Pv) infection showing enrichment 48 h post-infection (PI) of **(A)** *Total bacteria* (16S): *p* ≤ 0.002/4-6DPI, *p* ≤ 0.0004/8-10DPI; **(B)** *Elizabethkingia p* ≤ 0.001/48hPI, *p* ≤ 4.69E-05/72hPI, *p* ≤ 0.001/4-6DPI, *p* ≤ 0.0003/8-10DPI; **(C)** (*Serratia p* ≤ 0.0001/48hPI, *p* ≤ 8.73E-05//72hPI, *p* ≤ 1.85E-05/4-6DPI, *p* ≤ 0.0004)/8-10DPI; and **(D)** *Pseudomonas*. DPI, days post-infection.

We observed a significant loss in the gut bacterial population in *Plasmodium*-infected mosquitoes which remained below the detection limit until 36 h (Figure 7A). However, surprisingly, after 36 h the total bacterial population followed a gradual enrichment to multifold level till 10 days of gut infection (Figure 7A). Interestingly, *Elizabethkingia* and *Serratia* also showed a similar pattern of enrichment, except *Pseudomonas* whose population level remains least affected (Figures 7B–D).

**Figure 8:**
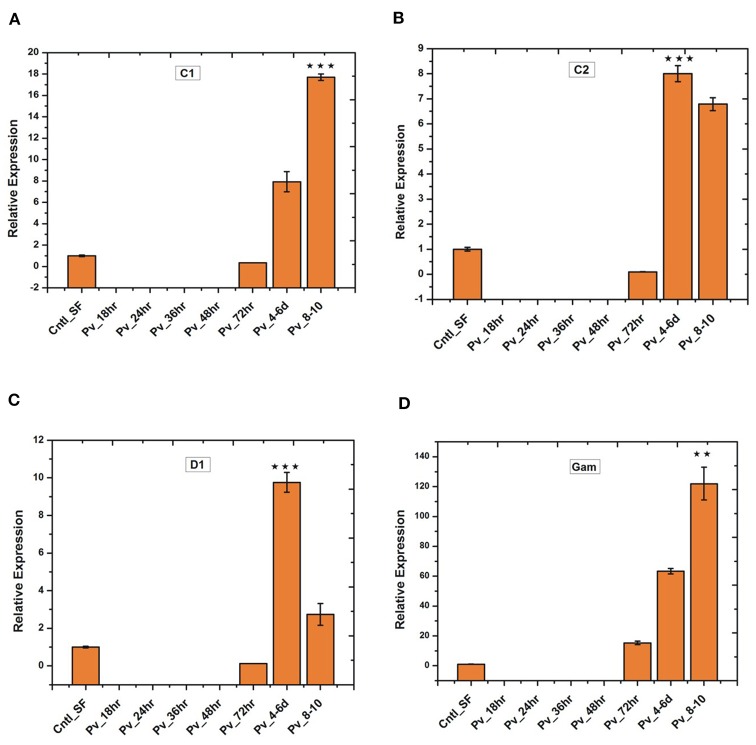
Relative quantification of gut immune transcripts in response to *Plasmodium vivax* infection: Transcriptional profiling of antimicrobial peptides (AMPs) C1 (*p* ≤ 9.00674E-05), C2 (*p* ≤ 0.0005), D1 (*p* ≤ 0.0008), Gambicin (*p* ≤ 0.002) showing early suppression of gut immunity which restored after 3 days of *P. vivax* infected blood meal. C1-C2, cecropin1 and cecropin2; D1, defensin1; Gam, Gambicin. **p < 0.005;* **p < 0.0005.

Since the blood meal-induced gut microbiota also boosts gut immunity, we tested whether *P. vivax* infection influences gut immune response. Time-dependent transcriptional profiling of all the selected anti-microbial peptides [also see (22)] showed a unique pattern of immunosuppression during the pre-invasive phase of ookinetes to early oocysts development (Figure 8). All the tested immune transcripts showed expression enrichment only 36 h post-infection. But, exceptionally, gambicin showed higher response than cecropin (C1, C2) and defensin (D1) (Figure 8), suggesting its unique role against late oocysts development of *P. vivax*.

### Laboratory Reared *Anopheles stephensi* Harbor *Wolbachia* Bacteria

Surprisingly, a qualified subset of 250 bp long metagenomic sequencing reads (6,532 blood-fed and 6,154 naïve mosquitoes gut) showed 100% identity to *Wolbachia* endosymbiont of *Chrysomya megacephala* (Accession #CP021120.1; Figure S6A, also see FASTA File S8). Also, identification of at least 7 mRNA transcripts, originating from distinct gut RNAseq libraries and encoding different *Wolbachia* homolog proteins (Figure S6B, Table ST7), further predicts the novel *Wolbachia* association. An ongoing similar comparative gut metagenomic analysis of Indian vector *A. culicifacies* (unpublished), reared in the same insectarium environment, did not yield a single sequence of *Wolbachia* origin, supporting that *A. stephensi* may exclusively harbor novel *Wolbachia* bacterial species.

## Discussion

Using a meta-transcriptomic strategy, we targeted to decode the molecular basis of tripartite gut-microbe-*P. vivax* interaction in the mosquito host *A. stephensi*. Our metagenomic study identifies *Elizabethkingia* and *Pseudomonas* as dominant gut-inhabiting bacteria in the laboratory-reared naïve adult female mosquitoes. In response to the blood meal, we observed a significant alteration of gut microbial community structure and enrichment of dominant bacterial species e.g., *Elizabethkingia* sp. (Flavobacteriales), *Pseudomonas* (Pseudomonadales), and *Serratia* (Enterobacteriales). Previous several studies have also reported a similar pattern of gut microbe enrichment (31, 32), but the nature of gut-microbe interactions, especially microbial proteins facilitating blood meal digestion, remains unclear (33). Available draft genome sequence of cultured bacterial species predicts several metabolic pathways, but no functional relation has been established (34, 35).

Functional annotation of at least ~391 *Elizabethkingia* transcripts identified from blood-fed mosquitoes gut-RNAseq data provide direct evidence of “*in vivo*” metabolically active proteins, which may have a role in blood meal digestion. Until 30 h of post-blood meal, an enriched expression of transcripts such as LEM-A, Ton-B dependent receptor, FecR, ABC transporter suggested their important role in iron metabolism (Table ST6). Possibly this is accomplished through siderophore uptake and oxidative stress management, a possible mechanism benefiting mosquito's survival and reproductive outcome (36–38).

It is known that gut endosymbionts also serve as potent modulators of sexual development and transmission of the malaria parasite in *Anopheles* mosquitoes (39, 40). This antagonistic relationship of gut bacteria has been observed in the sporogonic development of *Plasmodium* in several *Anopheline* mosquitoes (7, 40, 41). Introduction of *E. coli, Pseudomonas*, and *Serratia* by oral feeding reduces the gut oocyst load in *A. gambiae* (40), but species-specific interaction of the *Plasmodium* and bacteria remains unclarified. In our infectivity assay, we observed that *P. vivax* disables bacterial proliferation to keep an immunosuppression till invasion to the gut epithelium.

Though it is unknown how sexual stages of *Plasmodium* utilize ingested iron in the blood into the mosquito gut, an earlier study in *Anopheline* mosquitoes suggests that iron-depleted blood inhibits *P. falciparum* gametocyte activation, and hence the infectivity (42, 43). Thus, we hypothesized the first 24 h of gut-microbe-*Plasmodium* interaction in the gut lumen are crucial for *Plasmodium* survival, where it may limit the availability of iron/nutrients required for bacterial growth (44). Corroborating to earlier studies, we also observed that mosquitoes were able to restore the basal levels of gut microbiota within 30 h of uninfected blood meal digestion (6). However, surprisingly, *P. vivax* infection caused a major shift in gut microbiota restoration to an enriched state after 48 h of infection. Interestingly, this shift of bacterial enrichment boosted a similar pattern of gut immunity induction, till late oocysts exited gut epithelium (Figure 8). Together, we hypothesized that in the gut lumen, gut-microbe-*P. vivax* interaction undergoes a unique “flip-show” where an early suppression of gut bacteria may favor *Plasmodium* survival, but the late phase gut immunity activation may restrict gut oocysts population. A late phase anti-*Plasmodium* immunity has also been suggested in other mosquito-parasite interaction studies (45). Since we observed this pattern repeatedly for at least four independent experiments, thus it is very unlikely that it may be an undisclosed confounding effect of a blood sample originating from the patient having antibiotic treatment before diagnosis (46).

Paratransgenesis approaches for manipulating gut endosymbionts such as *Elizabethkingia, Serratia* to block parasite development are under progress (47–49). A dominant association of tested *Elizabethkingia, Pseudomonas, Serratia* with mosquito ovaries/eggs, and subsequent validation of transovarian transfer from F1, F2, and F3 generation (see Figure S7) supports an idea to select and target them for future manipulation. Alternatively, by manipulating intracellular endosymbiont such as *Wolbachia* induced male sterility and pathogen development inhibition, is rapidly gaining much attention for vector-borne disease control program (50, 51). Trial releases of *Wolbachia* inhabiting mosquitoes is now being proved as a tool to reduce dengue cases in several countries (52, 53). Laboratory validation of a similar strategy in *Anopheline* mosquitoes for malaria control is also in progress (54).

A surprising finding of at least ~6% metagenomic sequences and *Wolbachia* homolog protein-encoding transcripts, further established a natural association of a novel *Wolbachia* bacteria in laboratory-reared mosquitoes. Thus, we believe a systemic evaluation and validation of *Wolbachia* interaction influencing *Plasmodium* development and cytoplasmic incompatibility in *A. stephensi*, may be valuable to design a novel tool to fight malaria in India.

## Conclusion

**Figure 9:**
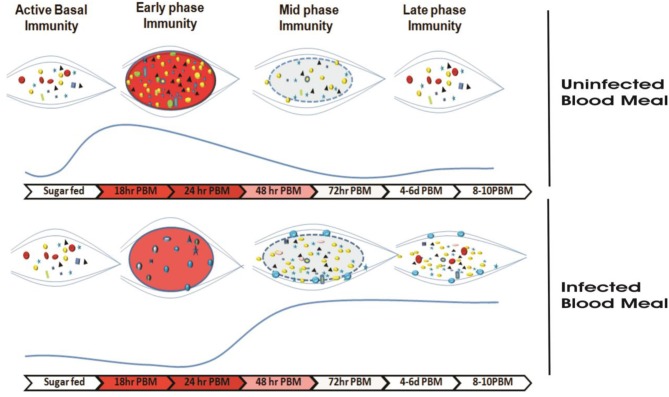
Survival strategy model of *P. vivax* during the pre-invasive phase of development inside the mosquito gut: A schematic representation of the microbial distribution in response to blood-feeding vs. *Plasmodium*-infected blood meal. In absence of *Plasmodium*, after a normal blood-feeding rapid proliferation of gut bacteria occurs in a nutrient-rich medium (of which Fe is indispensable to bacterial growth) during a time window of 18–24 h. Mosquito induces an innate immune response (via the production of various AMPs) to this incremental bacterial growth in order to tame the bacterial load and restore it to a basal level by 48 h. In presence of *Plasmodium, Plasmodium vivax* suppresses the proliferation of the gut bacteria possibly by altering Fe metabolism or nutrition physiology during initial hours (18–24 h) in a bid to dampen the mosquito innate immune response and to shore up ookinete invasion. In a direct competition for nutritional resources within the gut lumen between the parasite and bacteria, the parasite overcomes the bacteria. The parasite leaves the lumen and encysts beneath the basal lamina. After 48 h post-blood meal, in absence of competition with the parasite in the gut lumen allows the bacteria to proliferate possibly by feeding on undigested food left in the lumen. As the bacterial load rises, it leads to activation of mosquito innate immune responses followed by synthesis of various AMPs which not only limit the bacterial load but also limit the medium and late oocyst development. 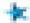, 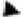, 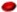, 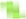, 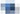, 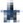, different bacteria residing the gut; 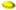, *Elizabethkingia*, 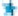, *Pseudomonas*, 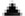, *Serratia*; 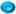, *Plasmodium viva;* 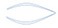, midgut; 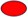, blood bolus after normal blood-feeding, 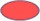, blood bolus after *Plasmodium*-infected blood uptake; 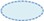, peritrophic matrix after blood digestion.

Several studies prove that immediately after blood feeding, a vital tripartite interaction occurs among mosquito-microbe-parasite in the mosquito's gut lumen. But the molecular basis that how *Plasmodium* manages its survival, development, and transmission is not well-known. For the first time, we establish that *P. vivax* causes an early suppression of gut microbial population, possibly by altering iron metabolism and nutritional physiology. And by this strategy, the parasite not only weakens gut immunity, but also favors successful invasion and development in the mosquito *A. stephensi* (Figure 9). With current data, we further propose that late oocysts/bursting oocysts releasing sporozoites alters gut bacterial susceptibility to boost late-phase immunity, a plausible mechanism to restrict the *Plasmodium* population [see (22); https://www.biorxiv.org/content/10.1101/774166v1].

## Data Availability Statement

The sequence data have been submitted to the NCBI SRA database under the following accession number: SAMN10496496- DNA_AS_SF_MG; SAMN10439711- AS_MG_BF_DNA for individual samples of metagenomics data described in the article. The sequences of Blood fed midgut RNASeq data is submitted with accession number-SRR8580010. All other data is included as Supplementary Material.

## Ethics Statement

The studies involving human participants were reviewed and approved by the Institutional Ethics Committee of NIMR (Ref#ECR/NIMR/EC/2012/41). The patients/participants provided their written informed consent to participate in this study.

## Author's Note

Successful malaria transmission relies on the competitive interactions of *Plasmodium* and mosquito's tissue-specific immune potential. Within 24 h of a blood meal, gut-microbiota grows exponentially and spikes a mosquito's immune response, which is detrimental to parasite development and survival. How *Plasmodium* manages to evade this pre-invasive immune barrier in the gut remains elusive. We investigated the influence of tripartite gut-microbiome-parasite interaction on human malaria parasite *Plasmodium vivax* in its natural/native vector *Anopheles stephensi*. Surprisingly, we found that infectious blood meal leads to dramatic suppression in the gut-bacteria population, a plausible strategy of *P. vivax* ookinetes to avoid immune responses. These findings provide a newer perspective on how *Plasmodium* may impact microbiota for its own survival. Disruption and manipulation of this gut-microbe-interaction may help to design new paratransgenesis molecular tools for malaria control.

## Author Contributions

PS, RD, and KP contributed idea and hypothesis generation, conceived, and designed the experiments. PS, CC, SK, JR, ST, TD, and DS contributed to design and performing the experiments, data acquisition, writing and editing. PS, KP, and RD data analysis and interpretation, data presentation, contributed reagents, materials, analysis tools, wrote, reviewed, edited, and finalized manuscript. All authors read and approved the final manuscript.

## Conflict of Interest

The authors declare that the research was conducted in the absence of any commercial or financial relationships that could be construed as a potential conflict of interest.

## References

[1] Attardo GM Hansen IA Raikhel AS. Nutritional regulation of vitellogenesis in mosquitoes: implications for anautogeny. Insect Biochem Mol Biol. (2005) 35:661–75. 10.1016/j.ibmb.2005.02.013 15894184

[2] Richards SL Anderson SL Yost SA. Effects of blood meal source on the reproduction of *Culex pipiens* quinquefasciatus (Diptera: Culicidae). J Vector Ecol. (2012) 37:1–7. 10.1111/j.1948-7134.2012.00194.x 22548531 PMC3342818

[3] Gaio ADO Gusmão DS Santos AV Berbert-Molina MA Pimenta PF Lemos FJ. Contribution of midgut bacteria to blood digestion and egg production in aedes aegypti (diptera: culicidae) (L.). Parasit Vectors. (2011) 4:105. 10.1186/1756-3305-4-105 21672186 PMC3125380

[4] Romoli O Gendrin M. The tripartite interactions between the mosquito, its microbiota and Plasmodium. Parasit Vectors. (2018) 11:200. 10.1186/s13071-018-2784-x 29558973 PMC5861617

[5] Pumpuni CB Demaio J Kent M Davis JR Beier JC. Bacterial population dynamics in three *Anopheline* species: the impact on *Plasmodium sporogonic* development. Am J Trop Med Hyg. (1996) 54:214–8. 10.4269/ajtmh.1996.54.214 8619451

[6] Das De T Sharma P Thomas T Singla D Tevatiya S Kumari S. Interorgan molecular communication strategies of local and systemic innate immune responses in mosquito *Anopheles stephensi*. Front Immunol. (2018) 9:148. 10.3389/fimmu.2018.00148 29515567 PMC5826171

[7] Dong Y Manfredini F Dimopoulos G. Implication of the mosquito midgut microbiota in the defense against malaria parasites. PLoS Pathog. (2009) 5:e1000423. 10.1371/journal.ppat.1000423 19424427 PMC2673032

[8] Smith RC Vega-Rodríguez J Jacobs-Lorena M. The Plasmodium bottleneck: malaria parasite losses in the mosquito vector. Mem Inst Oswaldo Cruz. (2014) 109:644–61. 10.1590/0074-0276130597 25185005 PMC4156458

[9] Rodgers FH Gendrin M Wyer CAS Christophides GK. Microbiota-induced peritrophic matrix regulates midgut homeostasis and prevents systemic infection of malaria vector mosquitoes. PLoS Pathog. (2017) 13:e1006391. 10.1371/journal.ppat.1006391 28545061 PMC5448818

[10] Noden BH Vaughan JA Pumpuni CB Beier JC. Mosquito ingestion of antibodies against mosquito midgut microbiota improves conversion of ookinetes to oocysts for *Plasmodium falciparum*, but not *P. yoelii*. Parasitol Int. (2011) 60:440–6. 10.1016/j.parint.2011.07.007 21763778 PMC3209551

[11] Simonetti AB. The biology of malarial parasite in the mosquito–a review. Mem Inst Oswaldo Cruz. (1996) 91:519–41. 10.1590/S0074-02761996000500001 9137738

[12] Chavshin AR Oshaghi MA Vatandoost H Pourmand MR Raeisi A Enayati AA. Identification of bacterial microflora in the midgut of the larvae and adult of wild caught *Anopheles stephensi*: a step toward finding suitable paratransgenesis candidates. Acta Trop. (2012) 121:129–34. 10.1016/j.actatropica.2011.10.015 22074685

[13] Chavshin AR Oshaghi MA Vatandoost H Pourmand MR Raeisi A Terenius O. Isolation and identification of culturable bacteria from wild *Anopheles culicifacies*, a first step in a paratransgenesis approach. Parasit Vectors. (2014) 7:419. 10.1186/1756-3305-7-419 25189316 PMC4261757

[14] Anglero-Rodriguez YI Blumberg BJ Dong Y Sandiford SL Pike A Clayton AM. A natural *Anopheles*-associated *Penicillium chrysogenum* enhances mosquito susceptibility to *Plasmodium* infection. Sci Rep. (2016) 6:34084. 10.1038/srep34084 27678168 PMC5039729

[15] Saraiva RG Kang S Simoes ML Anglero-Rodriguez YI Dimopoulos G. Mosquito gut antiparasitic and antiviral immunity. Dev Comp Immunol. (2016) 64:53–64. 10.1016/j.dci.2016.01.015 26827888

[16] Drexler AL Vodovotz Y Luckhart S. Plasmodium development in the mosquito: biology bottlenecks and opportunities for mathematical modeling. Trends Parasitol. (2008) 24:333–6. 10.1016/j.pt.2008.05.005 18603475 PMC2593109

[17] Bennink S Kiesow MJ Pradel G. The development of malaria parasites in the mosquito midgut. Cell Microbiol. (2016) 18:905–18. 10.1111/cmi.12604 27111866 PMC5089571

[18] Belachew EB. Immune response and evasion mechanisms of *Plasmodium falciparum* parasites. J Immunol Res. (2018) 2018:6529681. 10.1155/2018/6529681 29765991 PMC5889876

[19] Rosenberg R Wirtz RA Schneider I Burge R. An estimation of the number of malaria sporozoites ejected by a feeding mosquito. Trans R Soc Trop Med Hyg. (1990) 84:209–12. 10.1016/0035-9203(90)90258-G 2202101

[20] Ghosh AK Jacobs-Lorena M. Plasmodium sporozoite invasion of the mosquito salivary gland. Curr Opin Microbiol. (2009) 12:394–400. 10.1016/j.mib.2009.06.010 19608457 PMC2759692

[21] Simões ML Mlambo G Tripathi A Dong Y Dimopoulos G. Immune regulation of plasmodium is *Anopheles* species specific and infection intensity dependent. MBio. (2017) 8:e01631-17. 10.1128/mBio.01631-17 29042500 PMC5646253

[22] Tevatiya S Kumari S Chauhan C Singla D De TD Sharma P Genetic changes of *P. vivax* tempers host tissue-specific responses in *Anopheles stephensi*. bioRxiv. (2019) 774166 10.1101/774166 PMC9040150 35492403

[23] Sharma P Sharma S Mishra AK Thomas T Das De T Rohilla SL. Unraveling dual feeding associated molecular complexity of salivary glands in the mosquito *Anopheles culicifacies*. Biol Open. (2015) 4:1002–15. 10.1242/bio.012294 26163527 PMC4542284

[24] Sharma P Sharma S Maurya RK Das De T Thomas T Lata S. Salivary glands harbor more diverse microbial communities than gut in *Anopheles culicifacies*. Parasit Vectors. (2014) 7:235. 10.1186/1756-3305-7-235 24886293 PMC4062515

[25] Price MN Dehal PS Arkin AP. FastTree 2–approximately maximum-likelihood trees for large alignments. PLoS ONE. (2010) 5:e9490. 10.1371/journal.pone.0009490 20224823 PMC2835736

[26] Wang Q Garrity GM Tiedje JM Cole JR. Naive Bayesian classifier for rapid assignment of rRNA sequences into the new bacterial taxonomy. Appl Environ Microbiol. (2007) 73:5261–7. 10.1128/AEM.00062-07 17586664 PMC1950982

[27] Zhu YY Machleder EM Chenchik A Li R Siebert PD. Reverse transcriptase template switching: a SMART approach for full-length cDNA library construction. Biotechniques. (2001) 30:892–7. 10.2144/01304pf02 11314272

[28] Thomas T De TD Sharma P Lata S Saraswat P Pandey KC. Hemocytome: deep sequencing analysis of mosquito blood cells in Indian malarial vector *Anopheles stephensi*. Gene. (2016) 585:177–90. 10.1016/j.gene.2016.02.031 26915489

[29] Das De T Thomas T Verma S Singla D Chauhan C Srivastava V. A synergistic transcriptional regulation of olfactory genes drives blood-feeding associated complex behavioral responses in the mosquito *Anopheles culicifacies*. Front Physiol. (2018) 9:577. 10.3389/fphys.2018.00577 29875685 PMC5974117

[30] Dixit R Rawat M Kumar S Pandey KC Adak T Sharma A. Salivary gland transcriptome analysis in response to sugar feeding in malaria vector *Anopheles stephensi*. J Insect Physiol. (2011) 57:1399–406. 10.1016/j.jinsphys.2011.07.007 21787783

[31] Tchioffo MT Boissière A Abate L Nsango SE Bayibéki AN Awono-Ambéné PH. Dynamics of bacterial community composition in the malaria mosquito's epithelia. Front Microbiol. (2015) 6:1500. 10.3389/fmicb.2015.01500 26779155 PMC4700937

[32] Muturi EJ Dunlap C Ramirez JL Rooney AP Kim CH. Host blood-meal source has a strong impact on gut microbiota of *Aedes aegypti*. FEMS Microbiol Ecol. (2019) 95:fiy213. 10.1093/femsec/fiy213 30357406

[33] Chen S Blom J Walker ED. Genomic, physiologic, and symbiotic characterization of serratia marcescens strains isolated from the mosquito *Anopheles stephensi*. Front Microbiol. (2017) 8:1483. 10.3389/fmicb.2017.01483 28861046 PMC5561391

[34] Kukutla P Lindberg BG Pei D Rayl M Yu W Steritz M. Draft genome sequences of elizabethkingia anophelis strains R26T and Ag1 from the midgut of the malaria mosquito *Anopheles gambiae*. Genome Announc. (2013) 1:e01030-13. 10.1128/genomeA.01030-13 24309745 PMC3853068

[35] Pei D Hill-Clemons C Carissimo G Yu W Vernick KD Xu J. Draft genome sequences of two strains of *Serratia* spp. from the midgut of the malaria mosquito *Anopheles gambiae*. Genome Announc. (2015) 3:e00090-15. 10.1128/genomeA.00090-15 25767231 PMC4357753

[36] Köster W. ABC transporter-mediated uptake of iron, siderophores, heme and vitamin B12. Res Microbiol. (2001) 152:291–301. 10.1016/S0923-2508(01)01200-1 11421276

[37] Mettrick KA Lamont IL. Different roles for anti-sigma factors in siderophore signalling pathways of *Pseudomonas aeruginosa*. Mol Microbiol. (2009) 74:1257–71. 10.1111/j.1365-2958.2009.06932.x 19889096

[38] Wang R Xu H Du L Chou SH Liu H Liu Y. A TonB-dependent receptor regulates antifungal HSAF biosynthesis in Lysobacter. Sci. Rep. (2016) 6:26881. 10.1038/srep26881 27241275 PMC4886534

[39] Weiss B Aksoy S. Microbiome influences on insect host vector competence. Trends Parasitol. (2011) 27:514–22. 10.1016/j.pt.2011.05.001 21697014 PMC3179784

[40] Tchioffo MT Boissiere A Churcher TS Abate L Gimonneau G Nsango SE. Modulation of malaria infection in *Anopheles gambiae* mosquitoes exposed to natural midgut bacteria. PLoS ONE. (2013) 8:e81663. 10.1371/annotation/d8908395-a526-428c-b9ed-4430aaf8f7d7 24324714 PMC3855763

[41] Pumpuni CB Beier MS Nataro JP Guers LD Davis JR. *Plasmodium falciparum*: inhibition of sporogonic development in *Anopheles stephensi* by gram-negative bacteria. Exp Parasitol. (1993) 77:195–9. 10.1006/expr.1993.1076 8375488

[42] Ke H Sigala PA Miura K Morrisey JM Mather MW Crowley JR. The heme biosynthesis pathway is essential for *Plasmodium falciparum* development in mosquito stage but not in blood stages. J Biol Chem. (2014) 289:34827–37. 10.1074/jbc.M114.615831 25352601 PMC4263882

[43] Ferrer P Vega-Rodriguez J Tripathi AK Jacobs-Lorena M Sullivan DJ. Antimalarial iron chelator FBS0701 blocks transmission by *Plasmodium falciparum* gametocyte activation inhibition. Antimicrob Agents Chemother. (2015) 59:1418–26. 10.1128/AAC.04642-14 25512427 PMC4325789

[44] Clark MA Goheen MM Cerami C. Influence of host iron status on *Plasmodium falciparum* infection. Front Pharmacol. (2014) 5:84. 10.3389/fphar.2014.00084 24834053 PMC4018558

[45] Clayton AM Dong Y Dimopoulos G. The *Anopheles* innate immune system in the defense against malaria infection. J Innate Immun. (2014) 6:169–81. 10.1159/000353602 23988482 PMC3939431

[46] Gendrin M Rodgers FH Yerbanga RS Ouédraogo JB Basáñez MG Cohuet A. Antibiotics in ingested human blood affect the mosquito microbiota and capacity to transmit malaria. Nat Commun. (2015) 6:5921. 10.1038/ncomms6921 25562286 PMC4338536

[47] Chen S Bagdasarian M Walker ED. Elizabethkingia anophelis: molecular manipulation and interactions with mosquito hosts. Appl Environ Microbiol. (2015) 81:2233–43. 10.1128/AEM.03733-14 25595771 PMC4345385

[48] Wang S Dos-Santos ALA Huang W Liu KC Oshaghi MA Wei G. Driving mosquito refractoriness to *Plasmodium falciparum* with engineered symbiotic bacteria. Science. (2017) 357:1399–402. 10.1126/science.aan5478 28963255 PMC9793889

[49] Koosha M Vatandoost H Karimian F Choubdar N Oshaghi MA. Delivery of a genetically marked serratia AS1 to medically important arthropods for use in RNAi and paratransgenic control strategies. Microb Ecol. (2018) 78:185–94. 10.1007/s00248-018-1289-7 30460544

[50] Bourtzis K Dobson SL Xi Z Rasgon JL Calvitti M Moreira LA. Harnessing mosquito-Wolbachia symbiosis for vector and disease control. Acta Trop. (2014) 132(Suppl.):S150–63. 10.1016/j.actatropica.2013.11.004 24252486

[51] Jayakrishnan L Sudhikumar AV Aneesh EM. Role of gut inhabitants on vectorial capacity of mosquitoes. J Vector Borne Dis. (2018) 55:69–78. 10.4103/0972-9062.242567 30280704

[52] Dorigatti I McCormack C Nedjati-Gilani G Ferguson NM. Using *Wolbachia* for dengue control: insights from modelling. Trends Parasitol. (2018) 34:102–13. 10.1016/j.pt.2017.11.002 29183717 PMC5807169

[53] Garcia GA Sylvestre G Aguiar R da Costa GB Martins AJ Lima JBP. Matching the genetics of released and local *Aedes aegypti* populations is critical to assure *Wolbachia* invasion. PLoS Negl Trop Dis. (2019) 13:e0007023. 10.1371/journal.pntd.0007023 30620733 PMC6338382

[54] Bian G Joshi D Dong Y Lu P Zhou G Pan X. Wolbachia invades *Anopheles stephensi* populations and induces refractoriness to *Plasmodium* infection. Science. (2013) 340:748–51. 10.1126/science.1236192 23661760

