# Supplementary material for: Altered Gut Microbiota and Immunity Defines Plasmodium vivax Survival in Anopheles stephensi
Source: Front Immunol. 2020 May 14;11:609. doi: 10.3389/fimmu.2020.00609 (PMC7240202; doi:10.3389/fimmu.2020.00609)
Supplement: Supplementary file 3 [file Table_3.DOCX]

**S1: Technical work plan to decode molecular complexity of mosquito-microbiota-parasite interaction. In this study the two way approach including the metatranscriptomics based bioinformatics study and the Real time based relative abundance of the selected bacteria and related genes is followed to find the role of the residing bacteria in blood feeding and parasite transmission.**

**S2: Agarose Gel Picture showing the quality check of gDNA of the sugar fed *Anoph­­eles stephensi* midgut. First lane is the marker and 2^nd^ lane is of AS_SF_MG sample.**

**S3: The electropherogram of the Bioanalyzer 2100 analysis for the amplicon library which was purified by 1X AMpureXP beads showing the major peak within 500bp and 700 bp that was taken for subsequent bacterial sequencing, profiling and analysis.**

**S4: Real-time PCR based estimation of relative abundance of gut bacterial population in response to blood feeding. The figure shows the highest 16S rRNA expression post 24 hrs of blood feeding as compared to sugar feeding and early blood fed stages. At 48hrs PBM abundance of the bacteria retains at a level comparable to its sugar fed stage.**

**S5: Graphical representation of the diversity indices (a) Chao1 (b) Shannon alpha-diversity rarefaction curves of the sugar fed and blood fed midgut microbiomes of the *Anopheles* *stephensi* showing full extent of phylotype richness and quantifiable diversity estimation.**

**S6a: A 250 bp long Metagenomic reads NCBI/BLASTn analysis against NR database identifies Wolbachia endosymbiont sequnce in the mosquito *Anopheles stephensi* gut.**

**S6b: RNAseq based BLASTx analysis of putative trancripts predicts Wolbachia endosymbiont homolog proteins in the mosquito *Anopheles stephensi* gut.**

**S7: Relative quantity of different bacteria in the *Anopheles* *stephensi* ovary and the eggs of subsequent generations. In this figure the relative abundances of the selected bacteria viz. *Elizabethkingia*, *Serriatia* and *Pseudomonas* in the ovary of the parent generation and then first batch of eggs of the subsequent generations of the mosquito (F1, F2, F3) were relatively quantified.**

**S8: Fasta sequences of the Wolbachia sequences retrieved from the metagenomics data in the sugar fed and blood fed mosquito midgut.**

ST-1: Primer sequences used for the amplification of the V3-V4 hypervariable region of 16S rDNA gene of Eubacteria and Archaea for the 16S metagenomic library preparation

**ST-2 List of the primers used in the Real time and RT- PCR**

**ST-3: Comparative analysis of metagenomics data of the sugar and blood fed mosquito guts (Taxas has been classified at order level)**

**ST-4: Blood fed mosquitoes gut RNAseq database assembly and NR homology search analysis statistics**

**ST-5: Blood fed mosquito gut RNASeq database analysis against NR database to identify species homology *.***

**ST-6: Table showing the putative function of bacterial (EK) genes present in the *Anopheles stephensi* transcriptome and showed enriched expression in response to blood feeding.**

**ST-7 Details of the *Wolbachia* transcript coding protein retrieved from the different transcriptomes of *Anopheles stephensi* midgut.**
