# Supplementary material for: Altered Gut Microbiota and Immunity Defines Plasmodium vivax Survival in Anopheles stephensi
Source: Front Immunol. 2020 May 14;11:609. doi: 10.3389/fimmu.2020.00609 (PMC7240202; doi:10.3389/fimmu.2020.00609)
Supplement: Supplementary file 5 [file Data_Sheet_2.pdf]

Altered Gut Microbiota and Immunity Defines *Plasmodium vivax* Survival in *Anopheles stephensi*

Punita Sharma[1], Jyoti Rani[1,2], Charu Chauhan[1], Seena Kumari [1], Sanjay Tevatiya[1],Tanwee Das De[1], Deepali Savargaonkar[1], Kailash C. Pandey [1] and Rajnikant Dixit[1]*

1 Laboratory of Host-Parasite Interaction Studies, ICMR-National Institute of Malaria Research, New Delhi, India, 2 Bio and nanotechnology Department, Guru Jambheshwar University of Science and technology, Haryana, India

Supplemental Data Sheet 2

ST 1: Primer sequences used for the amplification of the V3-V4 hypervariable region of 16S rDNA gene of Eubacteria and Archaea for the 16S metagenomic library preparation

Sr.No.	Oligo Name	Oligo Sequence (5' to 3')	Length of primer	Product size (Approx.)
1	V3-Forward	CCTACGGGNGGCWGCAG	17	~ 460 bps
	V4-Reverse	GACTACHVGGGTATCTAATCC	21	

ST-2 List of the primers used in the Real time and RT- PCR

Sr. No.	Primer name	Forward Primer sequence (5' to 3')	Reverse Primer sequence (5' to 3')
1	Elizabethkingia	CGAGCGGTAGAGATTCTTCGG	ACACGTAAGTAGGTTTATCCCCAGA
2	Pseudomonas	GACGGGTGAGTAATGCCTA	CACTGGTGTTCCTTCCTATA
3	Serratia	CTGTCGTCAGCTCGTGTTGT	TTCATGGAGTCGAGTTGCAG
4	16s	TTGGAGAGTTTGATCCTGGCTC	ACGTCATCCCCACCTTCCTC
5	C1	AACCAACCGAACCGTATCAA	TTTCTCAGCTGCCTTGAACA
6	C2	AAGCTGCTCTTTCTCGTTGC	GTGAGGTACGCCCTATCCAA
7	D1	GATGAACTGCCCGAAGAGAC	TTGCTGGCTGTTGCAGTATC
8	Gambicin	ACTGTGGCTACGGGTACGTC	GCTTGTTCTTCCGGTGTGAT
9	Lem A	CGTTTACCAGAAACGTGCAA	TGCTGGTCTGCCTTTAGGTT
10	FECR	TTTCTTCCGGCTTTTGGATA	AAATATTGCAGTCCCGCTTG
11	ABC transporter	TTGTATCGATCATGGGGTCA	TCTTTTCGGGAAACATTCTGA
12	Sus C	TAGATGCGAACGGACTTCCT	CGGTTCCATCAGCAACTACA
13	Ton B	CATTGGGAAAGTAGGCGTGT	GACTGGATCCTGGCTTACCA

ST4: Blood fed mosquitoes gut RNAseq database assembly and NR homology search analysis statistics

Description	ASMGC
Total No. of bases	35,072,112
No. of Contigs	74,548
Total no. of transcripts	11159
Average Transcript Size	470
N50	539
Max Transcript Size	5,536
Total CDS analyzed (Post duplicate removal)	7,381
Best match to NR database	5041
Insect homolog sequences	4,562
Unknown/Hypt	2340
Non insect microbial	479

ST-6: Table showing the putative function of bacterial (EK) genes present in the *Anopheles stephensi* transcriptome and showed enriched expression in response to blood feeding.

Gene	Putative function
LemA	Quoram sensing, biofilm production,interspecies communication and siderophore production
Ton B,<br>FecR,<br>ABC transporter	Siderophore uptake and iron metabolism
SusC/RagA	import large degradation products of proteins (e.g. RagA) or carbohydrates (e.g. SusC) as nutrients

ST-7 Details of the *Wolbachia* transcript coding protein retrieved from the different transcripptomes of *Anopheles stephensi* midgut.

Sr. no.	Transcript label	Origin	Size (bp)	Coding nature	Percentage identity
1.	CDS_1174_Transcript_250	AS_MG_SF	1056	hypothetical protein[Wolbachia endosymbiont of Drosophila ananassae]	51.98%
2	CDS_3077_Trasncript_20360	AS_MG_BF	546	hypothetical protein [Wolbachia endosymbiont of Drosophila ananassae]	43.81%
3	CDS_7405_Trasncript_21832	AS_MG_PV_6-8D	354	hypothetical protein [Wolbachia pipientis]	46.15%
4	CDS_7433_Trasncript_21981	AS_MG_PV_6-8D	396	hypothetical protein [Wolbachia endosymbiont of Drosophila simulans]	31.88%
5	CDS_11533_Trasncript_48370	AS_MG_PV_6-8D	342	hypothetical protein [Wolbachia endosymbiont of Drosophila simulans]	43.01%
6	CDS_698_Trasncript_4595	AS_MG_PV_10D	1092	hypothetical protein [Wolbachia endosymbiont of Drosophila simulans]	32.45%
7	CDS_5445_Trasncript_19039	AS_MG_PV_10D	426	hypothetical protein [Wolbachia pipientis]	33.33%
